# Rare Diagnosis of Localized Breast Amyloidosis in the Setting of Lymphoplasmacytic Lymphoma

**DOI:** 10.31486/toj.25.0017

**Published:** 2025

**Authors:** Matthew Koury, Karthik Shankar, Walter M. Klein, Zonera Ali

**Affiliations:** ^1^ Department of Internal Medicine, Lankenau Medical Center, Wynnewood, PA; ^2^ Department of Hematology and Oncology, Lankenau Medical Center, Wynnewood, PA; ^3^ Department of Pathology, Bryn Mawr Hospital, Bryn Mawr, PA

**Keywords:** *Amyloid*, *amyloidosis*, *breast*, *lymphoma*

## Abstract

**Background:**

Localized amyloidosis of the breast is a rare diagnosis that is most often found incidentally during screening breast examinations or on imaging. This diagnosis can be associated with systemic amyloidosis or malignancy, making further investigation a necessity. Rarely, localized amyloidosis of the breast has been associated with lymphoplasmacytic lymphoma.

**Case Report:**

A female in her early 70s presented with a mass noted on breast examination. After biopsy, she was diagnosed with localized amyloidosis of the breast. After bone marrow biopsy, the patient was diagnosed with lymphoplasmacytic lymphoma. No evidence of systemic amyloidosis was found. Because the patient is asymptomatic, she is not undergoing treatment, but she is monitored clinically every 6 months.

**Conclusion:**

Breast amyloidosis is a rare diagnosis that should be considered when patients present with a breast mass. This case emphasizes the importance of a full diagnostic workup when breast amyloidosis is diagnosed to rule out systemic amyloidosis or an associated malignancy.

## INTRODUCTION

Amyloidosis is a disease characterized by extracellular deposits of abnormally configured protein into tissue. Amyloidosis can be localized to a single organ or can be systemic and cause devasting effects, particularly to the heart, kidneys, and nervous system, but amyloid deposits can affect nearly every organ.^1^ Breast amyloidosis is a particularly rare type of amyloidosis that has predominantly been reported in case reports and case series.^2-5^ Breast amyloidosis is most often diagnosed incidentally when a palpable mass is discovered or a breast screening examination raises concern for a tumorlike mass. Diagnosis is confirmed by biopsy and histologic examination. Most often, the specific type of amyloidosis seen in breast amyloidosis is light chain–associated amyloidosis (AL amyloidosis).^2^

The importance of making the diagnosis of breast amyloidosis is the association with malignancy and systemic amyloidosis. In a case series by Duckworth et al, nearly 40% of breast amyloidosis cases were associated with lymphoma.^3^ We identified 3 patients in the literature who specifically had lymphoplasmacytic lymphoma associated with breast amyloidosis.^3-5^ Consequently, when histopathology identifies breast amyloidosis, further workup is necessary to rule out systemic amyloidosis and malignancies because both conditions require treatment.

We present the case of a patient who was asymptomatically diagnosed with amyloidosis of the breast after a screening mammogram. Further workup and bone marrow biopsy revealed that the patient had associated lymphoplasmacytic lymphoma.

## CASE REPORT

A female in her early 70s with a medical history of hypertension, diabetes mellitus with peripheral neuropathy, and gastroesophageal reflux disease presented to the oncology office after her screening mammogram revealed nodular asymmetry of the right breast and calcifications in the right breast. These findings were new compared to an unremarkable screening mammogram 1 year prior. She was asymptomatic, with no night sweats, fevers, chills, or unexpected weight loss. No lymphadenopathy was palpable on physical examination. Her complete blood count was unremarkable and did not indicate cytopenia.

One week later, core needle biopsy of the right breast revealed scattered deposits of amorphous eosinophilic material within fibrocollagenous stroma, blood vessels, and ducts with calcifications. The histologic findings, combined with positive Congo red staining showing characteristic apple-green birefringence under polarized light, were diagnostic of amyloidosis (Figure). Apple-green birefringence is a hallmark feature of amyloid deposits under polarized microscopy. Further studies investigating the peptide profile confirmed lambda-type AL amyloidosis.

**Figure.**
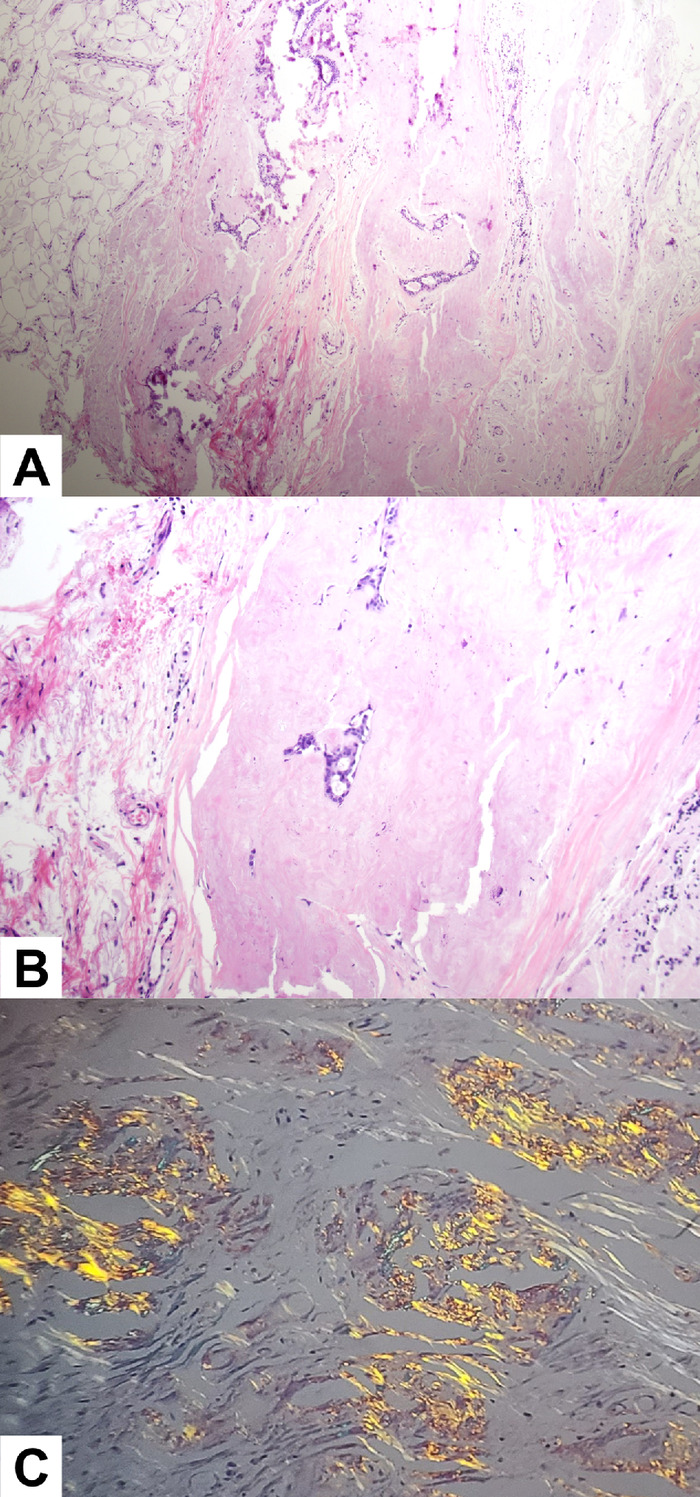
Pathology slides of the core needle biopsy reveal benign breast ducts surrounded by amorphous eosinophilic material consistent with amyloid: (A) magnification ×40, (B) magnification ×100. (C) Congo red staining shows apple-green birefringence.

After the diagnosis of amyloidosis was made, a multiple myeloma workup was performed that revealed free light chains with elevated lambda levels, and serum protein electrophoresis showed an M protein spike of 0.14 g/dL. Immunofixation identified a monoclonal immunoglobulin M kappa protein. Because of these findings, a bone marrow biopsy was performed that revealed mildly hypercellular bone marrow (approximately 40% cellular) with trilineage hematopoiesis. Flow cytometric analysis of the bone marrow aspirate revealed a small kappa-predominant B cell population (3.2%) and a small kappa-predominant plasma cell population (0.1%). The MYD88 mutation was present, which is diagnostic for lymphoplasmacytic lymphoma.

Computed tomography scan of the chest, abdomen, and pelvis demonstrated a likely benign 7-mm solid pulmonary nodule (stable from 11 years prior) and no evidence of systemic amyloidosis. Needle localized excisional biopsy was performed to confirm clear margins. No residual amyloid was found; Congo red staining was negative.

Because the patient is currently asymptomatic, no interventions or treatments are being performed. The patient is monitored every 6 months with clinical evaluation and blood tests (including serum protein electrophoresis and free light chains test), and she undergoes annual imaging.

## DISCUSSION

Our patient presented after a screening breast examination showed calcifications, and she was found to have the rare condition of breast amyloidosis. Further imaging screening for systemic amyloidosis was unrevealing, but the monoclonal protein spike on immunofixation warranted a bone marrow biopsy that led to the underlying diagnosis of lymphoplasmacytic lymphoma and prompted continued surveillance. The discordance noted between the kappa-predominant lymphoplasmacytic lymphoma and lambda-type amyloidosis is unusual, suggesting complex pathophysiology that warrants further investigation. This case is consistent with the few previously described reports of breast amyloidosis associated with lymphoplasmacytic lymphoma.^3-5^ However, our patient did not have systemic amyloidosis noted on imaging unlike almost half of the patients with breast amyloidosis in the Said et al case series.^4^

Our patient's thorough workup ensured a valid diagnosis and the exclusion of systemic amyloidosis. Research on breast amyloidosis is lacking. Further research is needed to elucidate the connection between breast amyloidosis and lymphoplasmacytic lymphoma and to provide long-term morbidity and mortality data for these patients.

## CONCLUSION

This case contributes to the limited literature on breast amyloidosis. While the diagnosis of breast amyloidosis is straightforward, this case underscores the importance of including amyloidosis as a possible diagnosis for breast lesions. Further, clinicians should ensure that patients diagnosed with breast amyloidosis undergo a comprehensive workup to identify comorbidities such as systemic amyloidosis and associated malignancies. More research is needed to understand the connection between lymphoplasmacytic lymphoma and amyloidosis.

## Notes

*This article meets the Accreditation Council for Graduate Medical Education and the American Board of Medical Specialties Maintenance of Certification competencies for Patient Care and Medical Knowledge.*

## References

[1] Kim BM, Kim YJ, Hur H, You JK. Localized primary breast amyloidosis and 1-year changes in imaging: a case report. Radiol Case Rep. 2020;15(12):2637-2640. doi: 10.1016/j.radcr.2020.09.001 33088378 PMC7567917

[2] Röcken C, Kronsbein H, Sletten K, Roessner A, Bässler R. Amyloidosis of the breast. Virchows Arch. 2002;440(5):527-535. doi: 10.1007/s00428-001-0555-z 12021928

[3] Duckworth LA, Cotta CV, Rowe JJ, Downs-Kelly E, Komforti MK. Amyloid in the breast: retrospective review with clinicopathological and radiological correlation of 32 cases from a single institution. Histopathology. 2021;79(1):57-66. doi: 10.1111/his.14329 33404113

[4] Said SM, Reynolds C, Jimenez RE, Amyloidosis of the breast: predominantly AL type and over half have concurrent breast hematologic disorders. Mod Pathol. 2013;26(2):232-238. doi: 10.1038/modpathol.2012.167 23018872

[5] Lui PC, Pang LM, Hlaing TT, Tse GM. Tumorous amyloidosis of the breast associated with disseminated malignant lymphoma. J Clin Pathol. 2004;57(3):334-335. doi: 10.1136/jcp.2003.012062 PMC1770252 14990614

